# Interchanging Functionality Among Homologous Elongation Factors Using Signatures of Heterotachy

**DOI:** 10.1007/s00239-013-9540-9

**Published:** 2013-01-31

**Authors:** Ercan Cacan, James T. Kratzer, Megan F. Cole, Eric A. Gaucher

**Affiliations:** 1 School of Biology, Georgia Institute of Technology, Atlanta, GA USA; 2 School of Chemistry, Georgia Institute of Technology, Atlanta, GA USA; 3 Department of Biology, Gaziosmanpaşa University, Tokat, 60250 Turkey

**Keywords:** Functional divergence, Heterotachy, Covarion

## Abstract

Numerous models of molecular evolution have been formulated to describe the forces that shape sequence divergence among homologous proteins. These models have greatly enhanced our understanding of evolutionary processes. Rarely are such models empirically tested in the laboratory, and even more rare, are such models exploited to generate novel molecules useful for synthetic biology. Here, we experimentally demonstrate that the heterotachy model of evolution captures signatures of functional divergence among homologous elongation factors (EFs) between bacterial EF-Tu and eukaryotic eEF1A. These EFs are GTPases that participate in protein translation by presenting aminoacylated-tRNAs to the ribosome. Upon release from the ribosome, the EFs are recharged by nucleotide exchange factors EF-Ts in bacteria or eEF1B in eukaryotes. The two nucleotide exchange factors perform analogous functions despite not being homologous proteins. The heterotachy model was used to identify a set of sites in eEF1A/EF-Tu associated with eEF1B binding in eukaryotes and another reciprocal set associated with EF-Ts binding in bacteria. Introduction of bacterial EF-Tu residues at these sites into eEF1A protein efficiently disrupted binding of cognate eEF1B as well as endowed eEF1A with the novel ability to bind bacterial EF-Ts. We further demonstrate that eEF1A variants, unlike yeast wild-type, can function in a reconstituted in vitro bacterial translation system.

**Electronic supplementary material:**

The online version of this article (doi:10.1007/s00239-013-9540-9) contains supplementary material, which is available to authorized users.

Models of molecular evolution attempt to capture the manner by which biological sequences accumulate substitutions over evolutionary timescales (Whelan and Goldman 2001; Yang and Rannala 2012). For instance, one well-known model captures the biological reality that individual sites do not accept substitutions in a uniform manner—some sites in a gene sequence evolve slowly while other sites in the same gene evolve quickly (Felsenstein and Churchill 1996; Yang 1994). In another instance, an individual site can have different substitution rates at different times in a gene’s evolutionary history (Gaucher et al. 2002b). Such site-specific rate switching was described by Walter Fitch in this very journal more than 40 years ago to describe a particular evolutionary-based sequence pattern observed in proteins (Fitch 1971; Fitch and Markowitz 1970; Miyamoto and Fitch 1995). This model has since been generalized to accommodate similar patterns of substitutions and now goes by the terms heterotachy or Type-I functional divergence. Heterotachy specifically describes the observation that a single site in a gene can be slowly evolving in one portion of a phylogenetic tree while rapidly evolving in another portion of the same tree (Abhiman et al. 2006; Da et al. 2002; Gaucher et al. 2002b; Gu 2001; Huelsenbeck 2002; Kolaczkowski and Thornton 2008; Lockhart et al. 1998; Lopez et al. 2002; Pupko and Galtier 2002; Roure and Philippe 2011; Siltberg and Liberles 2002; Tuffley and Steel 1998; Wang et al. 2007; Wang et al. 2011; Wu and Susko 2011) (Fig. 1a). This implies that the selective constraints acting at a single site can be reciprocal. In a similar manner, reciprocal selective constraints can occur briefly in a gene’s evolutionary past while later returning to identical constraints. Such a pattern results in Conserved-But-Different (or Type-II functional divergence) whereby rapidly evolving sites are later constrained to have slower rates (Gu 2001; Lopez et al. 2002) (Fig. 1b). In total, these patterns have been observed in numerous gene families. Less clear, however, is an understanding of the precise selective constraints that produce these patterns. Some researchers have argued that patterns of heterotachy arise from either neutral or purifying selection (Lopez et al. 2002), while others have argued that such patterns may arise from adaptive selection due to functional divergence among homologous sequences (Gaucher et al. 2002a; Gaucher et al. 2001).

**Fig. 1:**
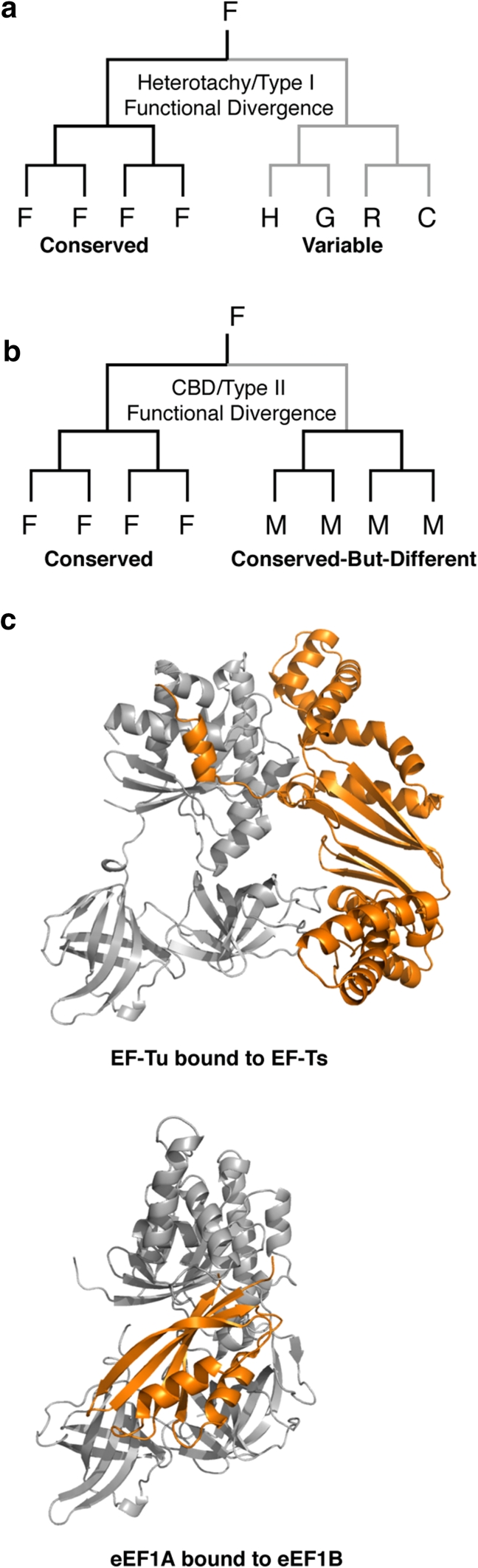
Signatures of functional divergence and structures of EFs. **a** Amino acid sequence pattern of a hypothetical site displaying heterotachy/Type-I functional divergence when analyzed within a phylogenetic framework. The selective constraints acting on the site are reciprocal between the two clades. *Dark branches* indicate strong selective constrains *while light branches* indicate relaxed constraints. **b** Amino acid sequence pattern of a hypothetical site displaying Type-II functional divergence when analyzed within a phylogenetic framework. The selective constraints acting on the site were briefly relaxed on an internal branch (*light branch*) such that one lineage mutated away from the original amino acid and this replacement was later fixed (also called Conserved-But-Different, *CBD*). **c** Schematic three-dimensional structures of bacterial EF-Tu (*grey*) bound to its nucleotide exchange factor EF-Ts (*top*, *orange*) and eukaryotic eEF1A (*grey*) bound to its nucleotide exchange factor eEF1B (*bottom*, *orange*). Although EF-Tu/eEF1A are homologous proteins, their respective exchange factors are not homologous (Color figure online)

Here we experimentally test whether sites displaying patterns of heterotachy/Type-I and Type-II may indeed be responsible for functional divergence among homologous proteins and whether they can be exploited to engineer protein function. An elongation factor (EF) gene family was chosen for experimental assays because previous studies have computationally predicted that sites displaying heterotachy are potentially involved in functional differences between bacterial and eukaryotic EFs (Gaucher et al. 2002a; Gaucher et al. 2001). The current study represents the first time that the heterotachy/Type-I and Type-II models of molecular evolution have been experimentally tested and is intended to accompany other experimental studies that tested models of molecular evolution (Bershtein and Tawfik 2008; Hillis et al. 1992; Merlo et al. 2007; Woods et al. 2006).

EF-Tu in bacteria and eEF1A in eukaryotes are homologous G-proteins that perform the same overall function in their respective domains of life. These biomolecules shuttle aminoacylated-tRNAs to the ribosome to participate in protein translation. Guanosine triphosphate (GTP) is hydrolyzed once the correct codon/anticodon base-pairing occurs thereby releasing the EF from the aminoacylated tRNA. The EF/guanosine diphosphate (GDP) complex is recharged to the active state through binding to a nucleotide exchange factor that replaces the spent nucleotide with a new GTP molecule.

Although EFs perform analogous functions in bacteria and eukaryotes, some particulars of their behaviors differ. Most notably, the manner by which nucleotide exchange occurs between bacterial and eukaryotic EFs is not homologous. Thus, EFs from eukaryotes have evolved a complementary mechanism of nucleotide exchange using eEF1B compared to their bacterial counterparts that use EF-Ts (Fig. 1c).

To determine the role heterotachy plays in carrying a signature of functional divergence among EFs, we have replaced residues in the yeast eEF1A with residues from the *E. coli* EF-Tu homolog. Sites were selected based on the strength and directionality of the signature of functional divergence between bacterial and eukaryotic EFs. The selection of sites had two objectives. On one hand, we wanted to determine if heterotachous sites overlap with the sites that enable the yeast eEF1A to bind its yeast nucleotide exchange factor - EF sites evolving slowly in eukaryotes but rapidly in bacteria. On the other hand, we wanted to determine if sites displaying functional divergence could be exploited to allow the yeast eEF1A to bind and interact with the *E. coli* nucleotide exchange factor - EF sites evolving rapidly in eukaryotes but slowly in bacteria.

## Results

The selection of sites for the two objectives is described below. First, for both objectives, sites were partitioned by their posterior probabilities (PP) of heterotachy/Type-I functional divergence and by rank-order for their signature of Type-II functional divergence (Gu 2001). Sites were then clustered into three nested groups: 1) heterotachy/Type-I sites having PP ≥90 %, 2) heterotachy/Type-I sites having PP ≥80 % and 3) heterotachy/Type-I sites having PP ≥80 % plus the top-ranked Type-II sites.

To determine if sites having signatures of functional divergence overlap with sites that govern eEF1A–eEF1B interactions, sites from the three groups above were retained if the distance between any atoms in eEF1A were within 5 Å of any atom in eEF1B. In addition, in order to be retained, Type-I EF sites had to exhibit a heterotachous pattern of slow evolution in eukaryotes but rapid evolution in bacteria. Such a pattern would be consistent with the notion that a site is slowly evolving in eukaryotes because it allows eEF1A to interact with eEF1B, whereas the homologous site in bacteria is rapidly evolving since it does not govern interactions between EF-Tu and EF-Ts. These variants are annotated as KnockOut 1 through 3 (KO1, KO2, KO3).

Reciprocally, in order to engineer the ability of eukaryotic eEF1A to bind to bacterial EF-Ts, sites from the three nested groups above were retained if the distance between residues on eEF1A were within 5 Å of any residue on EF-Ts (inferred from the structural alignment of EF-Tu and eEF1A bound to their respective nucleotide exchange factors). Type-I sites also had to exhibit a heterotachous pattern of slow evolution in bacteria but rapid evolution in eukaryotes. Such a pattern would be consistent with the notion that a site is slowly evolving in bacteria because it allows EF-Tu to interact with EF-Ts, whereas the homologous site in eukaryotes is rapidly evolving since it does not govern interactions between eEF1A and eEF1B. These variants are annotated as KnockIn 1 through 3 (KI1, KI2, KI3). Variants with both the set of KnockOut and KnockIn amino acid replacements were also synthesized for nested groups 2 and 3 and are annotated as KnockOut-KnockIn 2 and 3 (KOKI2 and KOKI3). A variant combining KnockOut-KnockIn residues from group 1 was not considered.

**Fig. 2:**
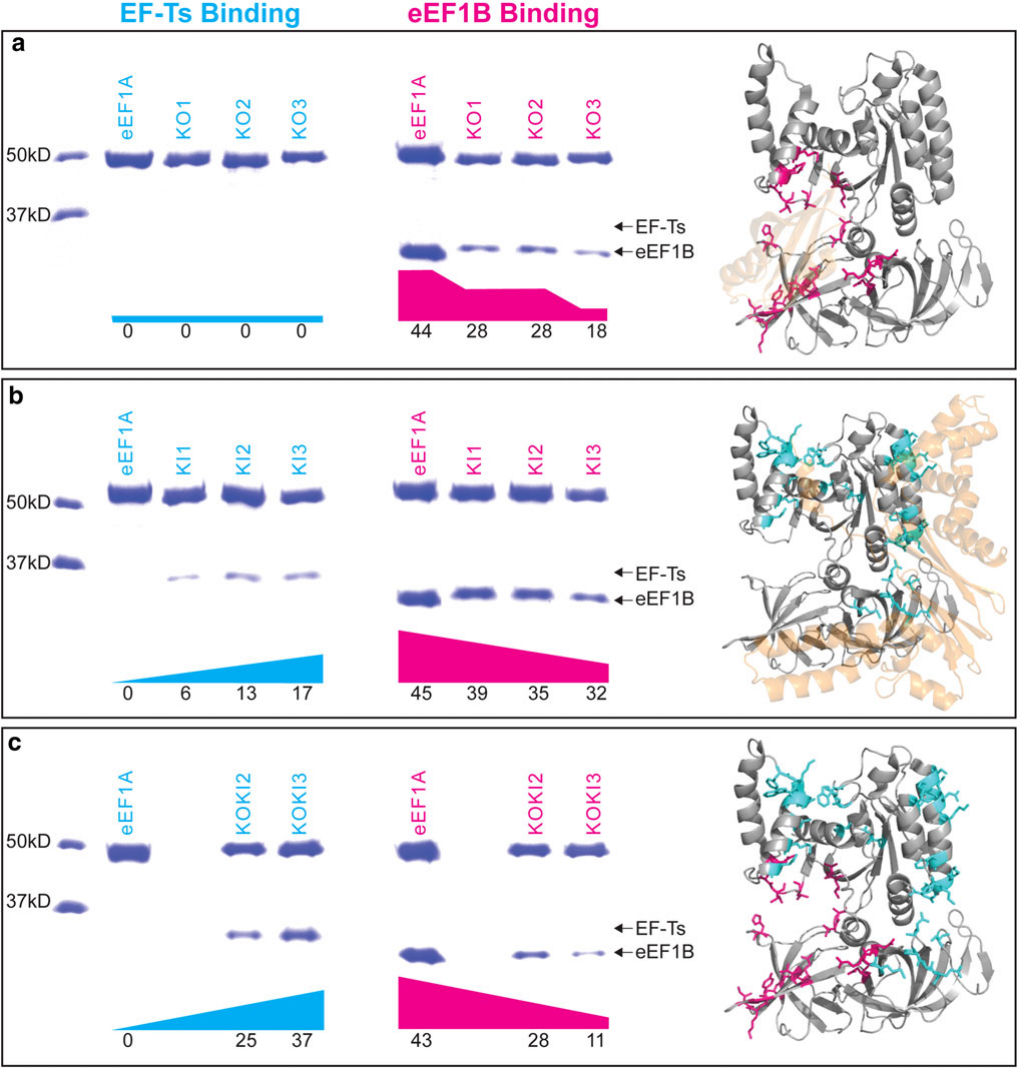
Loss of eukaryotic eEF1B binding and gain of bacterial EF-Ts binding in eEF1A variants. Binding assays were performed to measure the capacity of eEF1A variants to bind to bacterial EF-Ts and eukaryotic eEF1B.* Upper bands* correspond to eEF1A and variants while the *lower bands* correspond to nucleotide exchange factors. *Graphs below each gel image* represent the percentage of eEF1A bound by the nucleotide exchange factor (*cyan* for EF-Ts and* magenta* for eEF1B) and* numbers* below are the measured percentage band intensity for the lower (exchange factor) band compared to the total (exchange factor plus eEF1A band). **a** KnockOut variants KO1, KO2, and KO3. Crystal structure of eEF1A (*grey*) bound to eEF1B (*cream*) with the set of eEF1A residues corresponding to the set of KO3 mutations displayed in magenta. **b** KnockIn variants KI1, KI2, and KI3. Crystal structure of eEF1A (*grey*) superimposed with EF-Ts (*cream*) by structural alignment of eEF1A to EF-Tu bound to EF-Ts. The set of eEF1A residues corresponding to the set of KI3 mutations are displayed in* cyan*. **c** KnockOut-KnockIn variants KOKI2 and KOKI3. Crystal structure of eEF1A (*grey*) with the set of eEF1A residues corresponding to the set of KO3 mutations displayed in* magenta* and KI3 mutations displayed in* cyan* (the two residues present in both KO3 and KI3 are displayed in *cyan*) (Color figure online)

The eEF1A variants described above, as well as the native yeast eEF1A protein, were assayed for their abilities to bind the eukaryotic and bacterial nucleotide exchange factors (yeast eEF1B and *E. coli* EF-Ts). Figure 2 shows that the wild-type eEF1A protein binds eukaryotic eEF1B efficiently but cannot bind bacterial EF-Ts at a detectable level.

Figure 2a demonstrates that the KnockOut variants do indeed have reduced binding to eEF1B. KO1 and KO2 (PP ≥90 % heterotachous/Type-I sites and PP ≥80 % heterotachous/Type-I site, respectively) displayed substantially diminished ability to bind eEF1B compared to wild-type eEF1A. This nearly 50 % decrease in binding demonstrates that sites displaying heterotachous/Type-I patterns are indeed linked to functional divergence and that sites having the greatest affect lie in the PP range of 90–100 % but not 80–89 %. The KO3 variant demonstrated that sites displaying Type-II patterns are also responsible for functional divergence since this variant displayed an additional decrease in binding to eEF1B compared to KO2.

Figure 2b shows that the KnockIn variants have indeed acquired the ability to bind bacterial EF-Ts. All three variants from KI1 to KI3 displayed an incremental increase in their abilities to bind EF-Ts. This suggests that signals of functional divergence can vary in strength yet still shape biomolecular properties and that no single pattern dominates divergence among EF proteins. The KnockIn variants also had subtle decreases in their abilities to bind eEF1B. We suspect this diminished binding may have occurred due to the two sites that overlapped between the KI and KO variants despite the fact that the nucleotide exchange factors generally bind in different regions between the bacterial and eukaryotic EFs (compare structures in Fig. 2a, b, see Supplementary Figures S1–3 for a list of sites).

Figure 2c reveals some epistatic effects observed in the KnockOut-KnockIn combined variants. The KOKI variants displayed an enhanced ability to bind EF-Ts compared to the KI variants. This was an unexpected result because the KO variants did not have any ability to bind EF-Ts thus we would expect KOKI to have similar properties as KI. We have not fully addressed this difference but it does suggest that these KOKI sites have epistatic effects capable of enhanced binding to EF-Ts. Alternatively, the KOKI variants display similar diminished binding to eEF1B as the KO variants. This suggests that the combination of KO and KI sites had neither additive nor epistatic effects in regards to eEF1B binding.

To determine whether our selection of sites potentially biased the binding assays in the sense that we only focused on sites within 5 Å of their respective nucleotide exchange factors, a control eEF1A variant (eEF1A_C) was generated in which sites not displaying functional divergence but that fall within 5 Å of where EF-Ts would bind to eEF1A were selected and the *E. coli* EF-Tu residues were integrated into the yeast eEF1A protein. This eEF1A variant contained 17 residues from *E. coli* EF-Tu but still did not have any measurable binding activity to EF-Ts (Supplementary Figure S4). Thus, sites within the 5 Å cutoff itself were not sufficient to generate EF-Ts binding and thus highlights the importance of signals of heterotachy for efficient identification and manipulation of functional divergence.

The above results validate the connection between signals of heterotachy at the sequence level to binding interactions at the protein-level. The results do not, however, allow us to determine whether any particular function has been interchanged between EFs as it relates to protein translation. For instance, the abilities of variants KI3 and KOKI3 to bind EF-Ts does not necessarily indicate that these two eEF1A variants now have analogous functionalities as EF-Tu.

**Fig. 3:**
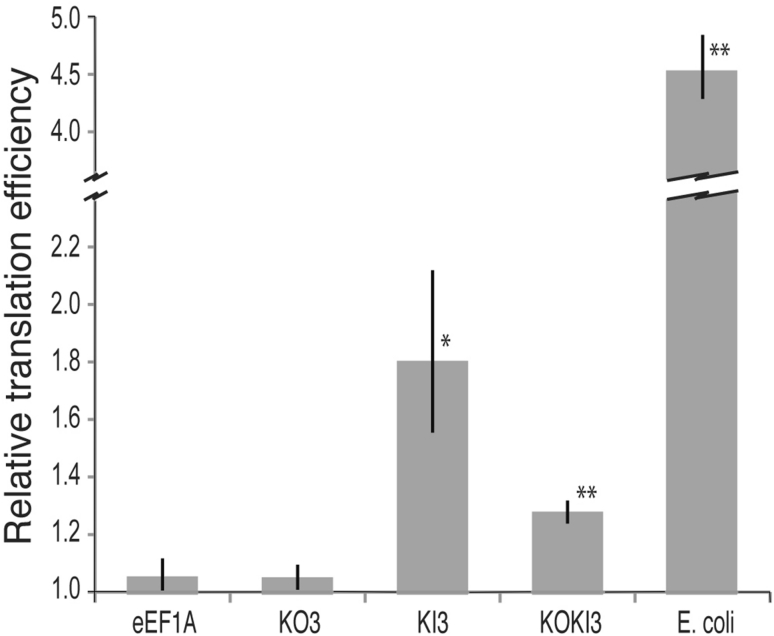
In vitro translation assay using wild-type eEF1A and the KOKI variants. A total of 15 replicate datasets were used from two independent protein purifications. Due to differences in protein refolding among the eEF1A proteins, a final buffer with 500 mM urea was used for the first round (with 1.3 mg of protein per reaction) and 125 mM urea for the second round (with 1 mg of protein per reaction). eEF1A variant KO3 was only active in the higher of the urea buffers, while KIKO3 was only active in the lower of the urea buffers. Data were collected using only the appropriate buffers for each of these two variants (6 replicates for KI3 and 9 replicates for KOKI3). All other analyses included data from both buffers to determine what, if any, affect the buffers have on the other variants (giving a total of 15 replicates). CPM were first normalized to the control reactions lacking any eEF1A protein and two data points were removed as statistical outliers (*p* < 0.05, Grubb’s test). Averages and standard errors of the mean are shown in the graph for each variant and * indicates significantly different from wild-type eEF1A at *p* < 0.05 while ** indicates significantly different at *p* < 0.01 using a student’s one-tailed *t* test. Results are shown relative to a background reaction lacking EF protein

We exploited a reconstituted in vitro protein translation system to address the functionality of these two eEF1A variants. This system is composed of recombinant *E. coli* biomolecules sufficient for in vitro translation (Shimizu et al. 2001). Such control of the system allows us to add/omit particular components. As such, the eEF1A variants could be added to the system in lieu of *E. coli* EF-Tu. Figure 3 demonstrates that both eEF1A variants KI3 and KOKI3 were able to participate significantly better in translation than the KO3 variant and the yeast wild-type eEF1A. Both KI3 and KOKI3 were able to bind EF-Ts (Fig. 2b, c) but the translation assay demonstrates that these variants have been engineered with the ability to actually participate in translation with bacterial components. It is curious that KOKI3 does not participate in translation as well as KI3 although the latter binds EF-Ts more efficiently. We suspect this may be due, in part, to the fact that these variants were soluble in different buffer systems. This may have affected the assays but additional studies will be required to dissect the differences as well as determine exactly how KI3 and KOKI3 are able to participate in translation while the other eEF1A proteins cannot.

## Discussion

We have experimentally demonstrated for the first time that particular patterns of functional divergence at the sequence level (specifically, heterotachy/Type-I and Type-II) are directly responsible for functional differences at the protein level between homologous proteins from bacteria and eukaryotes. This study provides a greater understanding of the molecular constraints that give rise to certain patterns identified using tools from the field of molecular evolution (Benner and Gaucher 2001; Levasseur et al. 2006; Liberles et al. 2012; Pagel and Meade 2008; Penn et al. 2008; Rasmussen and Kellis 2007; Studer and Robinson-Rechavi 2010; Wertheim et al. 2012; Whelan et al. 2011). In the near-term, our ability to connect heterotachous patterns to different levels of selection will be necessary (Blanquart and Lartillot 2008). For instance, heterotachous patterns may arise based on co-evolutionary constraints that maintain thermodynamic stability of a protein (Pollock et al. 2012). It would be improper to invoke functional divergence per se when heterotachous patterns arise under such a scenario.

Although it seems reasonable to conclude that functional divergence explains heterotachous patterns among EFs, it does raise the intriguing question as to how the patterns arose in the first place (we know why they have been maintained) – did adaptation shape the evolutionary trajectory of these sequence patterns or did intra-protein co-evolutionary biomolecular constraints guide EFs in a domain of life (either bacteria or eukaryotic) down a particular sequence trajectory and then evolution “dealt” with these sequence patterns which in turn gave rise to two disparate exchange factors (EF-Ts and eEF1B)? Interestingly, the EF family itself may provide insight between these two scenarios. We elected to not pursue a detailed analysis of the reciprocal set of experiments whereby we swap nucleotide exchange factor binding in EF-Tu because *E. coli*’s EF-Tu is inherently capable of binding yeast’s eEF1B exchange factor in a partial manner (this is in contrast to yeast’s eEF1A complete inability to bind *E. coli*’s EF-Ts as shown earlier). This may represent a type of vestigial property from when bacteria and eukaryotes diverged more than 3.5 billion years ago and whereby the ancestral EF was adapting toward or away from eEF1B binding. And one assumption is that residual binding between EF-Tu and eEF1B has not been fully purged due to overlapping co-evolutionary constraints.

Future studies will also have to determine how the strength of patterns of functional divergence at the sequence level accurately describes real divergence at the biomolecular level. The inclusion of sequences, phylogenetic accuracy, and models of molecular evolution necessarily influence signals of functional divergence at the sequence level. Some details of these signals are understood from a phylogenetic and protein perspective (Gaucher and Miyamoto 2005; Kolaczkowski and Thornton 2004; Liberles et al. 2012; Wang et al. 2011). However, more details are required before we understand the connection between sequence patterns and the biomolecular properties that selective or neutral forces shape as a consequence of the patterns themselves (Pollock et al. 2012; Studer and Robinson-Rechavi 2010).

We anticipate that our results provide rigor to evolutionary analyses and serve as a bridge between computational and experimental approaches. Additional studies will need to be conducted to determine whether heterotachy is widely associated with divergence at the biochemical level. In the other direction, we anticipate that our approach is useful to the synthetic biology field since we were able to create a chimeric protein endowed with expanded functionalities simply based on evolutionary sequence patterns. Such an approach could be exploited to generate synthetic systems having novel properties. In doing so, this would validate an evolutionary synthetic biology that combines natural evolution with protein engineering principles (Cole and Gaucher 2011a; Cole and Gaucher 2011b).

## Materials and Methods

### Bacterial Strains and Vectors

Tuner (DE3) pLysS competent cells (Novagen) were used for protein expression. The pET-15b (Novagen) amino-terminus His-tag vector was used for expression of the nucleotide exchange factors eEF1B and EF-Ts. The pET-21a (Novagen) carboxyl-terminus His-tag vector was used for expression of EFs EF-Tu, eEF1A, and eEF1A variants. The genes used in this study were synthesized by DNA2.0, Inc. and Genewiz, Inc.

### Expression and Purification of EF-Tu, EF-Ts, and eEF1B

A single bacterial colony was inoculated into 3 mL of Luria-Bertani (LB) media and incubated overnight at 37 °C with shaking. The overnight culture was diluted into a flask containing 250 mL of fresh LB media, 100 μg/mL carbenicillin, and 50 μg/mL chloramphenicol. The cells were grown at 37 °C to a density of 0.6 (A_600_) and then induced at a final concentration of 1 mM Isopropyl β-D-1-thiogalactopyranoside (IPTG). The culture was incubated at 37 °C for 4 h. The cells were collected by centrifugation at 4,500×*g* for 20 min. The wet weight of the pellet was determined and stored at −80 °C.

The frozen cells were thawed at room temperature for 15 min and re-suspended in BugBuster Protein Extraction Reagent (Novagen). The cell pellet was completely re-suspended using 5 mL BugBuster with 5 μL (25 units) of Benzonase (Novagen*)* per gram of wet cell pellet. The cell suspension was incubated on a shaking platform for 30 min at room temperature. The insoluble cell debris was removed by centrifugation at 10,000 × g for 30 min at 4 °C.

Ni–NTA columns were equilibrated with binding buffer (50 mM NaH_2_PO_4_, 500 mM NaCl, 5 mM MgCI_2_, and 5 mM imidazole, pH 7.6). The cleared lysate was loaded onto the equilibrated Ni–NTA column. The column was washed five times with wash buffer (50 mM NaH_2_PO_4_, 500 mM NaCl, 5 mM MgCI_2_, and 50 mM imidazole, pH 7.6). The protein was eluted with elution buffer (50 mM NaH_2_PO_4_, 500 mM NaCl, 5 mM MgCI_2_, and 500 mM imidazole, pH 7.6). The purified protein was dialyzed against 50 mM Tric-HCI (pH 7.8), 100 mM KCl, 1 mM DTT, and 3 mM MgCI_2_. In all steps, the flow-through was saved for analysis by SDS-PAGE to check the stringency of the conditions.

### Expression and Purification of eEF1A and Variants

The conditions for protein expression were identical as above. However, the cells were induced at a final concentration of 0.5 mM IPTG and the frozen cells were thawed at room temperature for 15 min and then re-suspended in 8 M urea, 100 mM NaH_2_PO_4_, 5 mM MgCI_2_, and 10 mM Tris–HCl (pH 8.0). Cell lysis was achieved by sonication and the suspension was incubated on a shaking platform for 2 h at room temperature. The cell debris was removed by centrifugation at 10,000×*g* for 30 min at room temperature.

The supernatant contained the denatured protein and was transferred to a Ni–NTA column equilibrated with denaturing binding buffer (8 M urea, 10 mM NaH_2_PO_4_, 5 mM MgCl_2_, and 10 mM Tris–HCl, (pH 8.0)). The column was washed five times with 8 M urea, 100 mM NaH_2_PO_4_, 5 mM MgCI_2_, 10 mM Tris–HCl, and 50 mM imidazole (pH 8.0). Elution occurred in 8 M urea, 100 mM NaH_2_PO_4_, 5 mM MgCI_2,_ 10 mM Tris–HCl, and 300 mM imidazole (pH 8.0). In all steps, the flow-through was saved for analysis by SDS-PAGE to check the stringency of the conditions.

### Refolding Denatured-Purified Proteins

Denatured purified eEF1A protein (and variants) was diluted two-fold in buffer consisting of 50 mM Tris–HCl, 20 mM NaCI, 100 mM KCI, (pH 8.2), and then stepwise dialyzed against 2 M urea, 50 mM Tris-HCI, 20 mM NaCI, 100 mM KCI, (pH 8.2); 1 M urea, 50 mM Tris-HCI, 20 mM NaCI, 100 mM KCI, (pH = 8.2); 150–500 mM urea, 50 mM Tris-HCI, 20 mM NaCI, 100 mM KCI, (pH = 8.2), respectively, using 20 kilodalton molecular weight cut-off dialysis cassette (Thermo Scientific). Each dialysis step lasted 12 h.

### Removal of His-Tag from EF-Ts and eEF1B

The His-tag was removed from nucleotide exchange factors using thrombin (Novagen). Thrombin was diluted in 1:25 thrombin dilution buffer (50 mM sodium citrate pH 6.5, 200 mM NaCl, 0.1 % PEG-8000, 50 % glycerol) and mixed with target protein and 10X thrombin cleavage buffer (200 mM Tris–HCl, 1.5 M NaCl, 25 mM CaCl_2_, (pH = 8.4)). The mixture was incubated at 25 °C with agitation for 16 h. Reactions were stopped with protease inhibitor complex and incubated for 1 h. To clear all His-tags and any potentially uncleaved His-tagged protein, the reaction was loaded onto an equilibrated Ni–NTA column. Flow-through was again passed over another equilibrated Ni–NTA column to confirm that all flow-through was free of His-tags. Samples were analyzed by SDS-PAGE and confirmed that all His-tags were cleaved and removed from the samples (Supplemental Figure S5).

### Protein Quantification

Proteins used in binding assays were quantified by performing Bradford protein assays. Each sample was measured in triplicate. One mL of Bradford solution (Bio-Rad) was incubated at room temperature for 30 min and followed by the addition of 20 μL of sample protein. After 5 min incubation at room temperature, samples were transferred to a cuvette and absorption at 595 nm was plotted against a bovine gamma globulin concentration curve.

### Pull-Down (Binding) Assay

His-tagged eEF1A and its variants were mixed with either eukaryotic (eEF1B) or bacterial (EF-Ts) nucleotide exchange factors (without His-tags) at a ratio of 1:1.5 and incubated at room temperature for 4 h in an incubation buffer of 55 mM Tris–HCl (pH 7.8), 130 mM KCl, 20 mM NaCI, and 2 mM EDTA. Control reactions were performed with wild-type EF-Tu and EF-Ts and with wild-type eEF1A and eEF1B to confirm proper folding of all individual reaction components. Before loading on a Ni–NTA column, samples were diluted two-fold in incubation buffer without EDTA. The dilution was done to prevent interference of EDTA with the column. The column was washed twice with buffer A containing 55 mM Tris-HCI (pH 8.2), 20 mM NaCI, 10 mM KCI, 500 mM urea, and 10 mM imidazole to remove nonspecific binding. Lastly, samples were eluted with buffer B containing 55 mM Tris-HCI (pH 8.2), 20 mM NaCI, 10 mM KCI, 500 mM urea, and 500 mM imidazole and analyzed by SDS-PAGE.

The binding efficiencies of eEF1A and the variants were determined by densitometric analyses using the ImageJ software package available from the National Institutes of Heath. The percentage of total-lane intensity contributed by the exchange factor band (EF-Ts or eEF1B) in each binding assay was used as a metric to compare binding efficiencies of eEF1A and the variants to the exchange factors.

### Statistical Analysis

DIVERGE software (Gu and Vander Velden 2002) was used to detect functional divergence among EF protein family members, based on site-specific rate shifts. A total of 30 EF-Tu and eEF1A protein sequences and a phylogenetic tree from our previous work (Gaucher et al. 2001) were analyzed. PP were calculated to determine whether a site may have experienced heterotachy/Type-I or Type-II patterns of divergence. As a test of robustness, we repeated the analysis with an expanded set of sequences. We calculated scores for Type-I and Type-II patterns using a set of 50 eukaryotic and 50 bacterial sequences combined from a set of phylogenetically diverse species. Of the 39 sites identified as exhibiting robust levels of functional diverence in our original analysis (Type-I sites having a cut-off of 90 % and the top Type-II sites) we again identified all but 5 of these sites. All 5 sites displayed a combination of Type-I and Type-II functional divergence that confounded the algorithms’ abilities to call the site as either Type-I or Type-II.

Pymol software was used to map the distribution of heterotachy/Type-I and Type-II sites across the three-dimensional structure of EF-Tu and eEF1A. In Pymol, the 1EFU (Kawashima et al. 1996) and 1F60 (Andersen et al. 2000) structures were aligned to identify sites that were within 5 Å of the opposite nucleotide exchange factor (i.e., sites on eEF1A within 5 Å of EF-Ts when EF-Tu and eEF1A bound to their respective exchange factors are structurally aligned). Sites to mutate were selected based on the DIVERGE analysis using parameters and cutoffs described in the main text.

### In Vitro Translation Reactions

Translation reactions were carried out using a customized in vitro translation assay kit (PURExpress, New England Biolabs, NEB), where the amino acids, tRNAs, and EF-Tu are added separately to a mix containing the remaining components necessary for translation. Reactions were in 6 μL total volume and contained 1 μL of solution A, 0.5 μL of amino acid mixture, 0.5 μL of tRNA, 1.3 μL solution B (all of the aforementioned supplied by NEB), then 0.1 μL of RNAse inhibitor (40,000 U/mL), 0.25 μL of S-35 methionine (Perkin-Elmer NEG009T), 0.2 μL of template DNA (250 ng/μL), and 1.95 μL of eEF1A proteins were added (see Fig. 3 for protein concentrations). A master mix of all the components minus the eEF1A proteins was assembled on ice and then dispensed to reaction tubes containing the eEF1A protein in its final refolding buffer. Template DNA was supplied on a plasmid containing a T7 promoter, ribosome binding site and a short gene that codes the peptide MVEVRHHHHHH. Reactions incubated for 3 h at 37 °C and were terminated by adding 50 μL of stop buffer (50 mM Tris–HCl pH 8.0, 300 mM NaCl, 5 mM βmercaptoethanol). Reactions were then transferred to 0.2 micron spin columns containing 15 μL of Ni–NTA agarose and rotated at room temperature for 1 h. Peptide product was collected after two washes with stop buffer by a 15-min elution at room temperature using 50 μL of 500 mM imidazole. 45 μL of the eluted peptide product was added to 2 mL of scintillation fluid and counts per minute (CPM) were measured.

## Electronic supplementary material

Below is the link to the electronic supplementary material.

Supplementary material 1 (PDF 2804 kb)

## References

[CR1] Abhiman S, Daub CO, Sonnhammer ELL (2006). Prediction of function divergence in protein families using the substitution rate variation parameter alpha. Mol Biol Evol.

[CR2] Andersen GR, Pedersen L, Valente L, Chatterjee I, Kinzy TG, Kjeldgaard M, Nyborg J (2000). Structural basis for nucleotide exchange and competition with tRNA in the yeast elongation factor complex eEF1A:eEF1Balpha. Mol Cell.

[CR3] Benner SA, Gaucher EA (2001). Evolution, language and analogy in functional genomics. Trends Genet.

[CR4] Bershtein S, Tawfik DS (2008). Ohno’s model revisited: measuring the frequency of potentially adaptive mutations under various mutational drifts. Mol Biol Evol.

[CR5] Blanquart S, Lartillot N (2008). A site- and time-heterogeneous model of amino acid replacement. Mol Biol Evol.

[CR6] Cole MF, Gaucher EA (2011). Exploiting models of molecular evolution to efficiently direct protein engineering. J Mol Evol.

[CR7] Cole MF, Gaucher EA (2011). Utilizing natural diversity to evolve protein function: applications towards thermostability. Curr Opin Chem Biol.

[CR8] Da L, Kumar VG, Tay A, Mamun AA, Ho WK, See A, Chan L (2002) Run-to-run process control for chemical mechanical polishing in semiconductor manufacturing. Proceedings of the 2002 Ieee International Symposium on Intelligent Control: pp. 740–745

[CR9] Felsenstein J, Churchill GA (1996). A Hidden Markov Model approach to variation among sites in rate of evolution. Mol Biol Evol.

[CR10] Fitch WM (1971). Rate of change of concomitantly variable codons. J Mol Evol.

[CR11] Fitch WM, Markowitz E (1970). An improved method for determining codon variability in a gene and its application to the rate of fixation of mutations in evolution. Biochem Genet.

[CR12] Gaucher EA, Miyamoto MM (2005). A call for likelihood phylogenetics even when the process of sequence evolution is heterogeneous. Mol Phylogenet Evol.

[CR13] Gaucher EA, Miyamoto MM, Benner SA (2001). Function-structure analysis of proteins using covarion-based evolutionary approaches: elongation factors. Proc Natl Acad Sci U S A.

[CR14] Gaucher EA, Das UK, Miyamoto MM, Benner SA (2002). The crystal structure of eEF1A refines the functional predictions of an evolutionary analysis of rate changes among elongation factors. Mol Biol Evol.

[CR15] Gaucher EA, Gu X, Miyamoto MM, Benner SA (2002). Predicting functional divergence in protein evolution by site-specific rate shifts. Trends Biochem Sci.

[CR16] Gu X (2001). Maximum-likelihood approach for gene family evolution under functional divergence. Mol Biol Evol.

[CR17] Gu X, Vander Velden K (2002). DIVERGE: phylogeny-based analysis for functional-structural divergence of a protein family. Bioinformatics.

[CR18] Hillis DM, Bull JJ, White ME, Badgett MR, Molineux IJ (1992). Experimental phylogenetics: generation of a known phylogeny. Science.

[CR19] Huelsenbeck JP (2002). Testing a covariotide model of DNA substitution. Mol Biol Evol.

[CR20] Kawashima T, Berthet-Colominas C, Wulff M, Cusack S, Leberman R (1996). The structure of the Escherichia coli EF-Tu.EF-Ts complex at 2.5 A resolution. Nature.

[CR21] Kolaczkowski B, Thornton JW (2004). Performance of maximum parsimony and likelihood phylogenetics when evolution is heterogeneous. Nature.

[CR22] Kolaczkowski B, Thornton JW (2008). A mixed branch length model of heterotachy improves phylogenetic accuracy. Mol Biol Evol.

[CR23] Levasseur A, Gouret P, Lesage-Meessen L, Asther M, Asther M, Record E, Pontarotti P (2006). Tracking the connection between evolutionary and functional shifts using the fungal lipase/feruloyl esterase A family. Bmc Evol Biol.

[CR24] Liberles DA, Teichmann SA, Bahar I, Bastolla U, Bloom J, Bornberg-Bauer E, Colwell LJ, de Koning APJ, Dokholyan NV, Echave J, Elofsson A, Gerloff DL, Goldstein RA, Grahnen JA, Holder MT, Lakner C, Lartillot N, Lovell SC, Naylor G, Perica T, Pollock DD, Pupko T, Regan L, Roger A, Rubinstein N, Shakhnovich E, Sjolander K, Sunyaev S, Teufel AI, Thorne JL, Thornton JW, Weinreich DM, Whelan S (2012). The interface of protein structure, protein biophysics, and molecular evolution. Protein Sci.

[CR25] Lockhart PJ, Steel MA, Barbrook AC, Huson DH, Charleston MA, Howe CJ (1998). A covariotide model explains apparent phylogenetic structure of oxygenic photosynthetic lineages. Mol Biol Evol.

[CR26] Lopez P, Casane D, Philippe H (2002). Heterotachy, an important process of protein evolution. Mol Biol Evol.

[CR27] Merlo LM, Lunzer M, Dean AM (2007). An empirical test of the concomitantly variable codon hypothesis. Proc Natl Acad Sci U S A.

[CR28] Miyamoto MM, Fitch WM (1995). Testing the covarion hypothesis of molecular evolution. Mol Biol Evol.

[CR29] Pagel M, Meade A (2008). Modelling heterotachy in phylogenetic inference by reversible-jump Markov chain Monte Carlo. Philos Transact R Soc B Biol Sci.

[CR30] Penn O, Stern A, Rubinstein ND, Dutheil J, Bacharach E, Galtier N, Pupko T (2008) Evolutionary modeling of rate shifts reveals specificity determinants in HIV-1 Subtypes. Plos Comput Biol 4(11):e1000214 10.1371/journal.pcbi.1000214 PMC2566816 18989394

[CR31] Pollock DD, Thiltgen G, Goldstein RA (2012). Amino acid coevolution induces an evolutionary Stokes shift. Proc Natl Acad Sci USA.

[CR32] Pupko T, Galtier N (2002). A covarion-based method for detecting molecular adaptation: application to the evolution of primate mitochondrial genomes. Proc R Soc Lond Ser B Biol Sci.

[CR33] Rasmussen MD, Kellis M (2007). Accurate gene-tree reconstruction by learning gene- and species-specific substitution rates across multiple complete genomes. Genome Res.

[CR34] Roure B, Philippe H (2011) Site-specific time heterogeneity of the substitution process and its impact on phylogenetic inference. Bmc Evol Biol 11:17 10.1186/1471-2148-11-17 PMC3034684 21235782

[CR35] Shimizu Y, Inoue A, Tomari Y, Suzuki T, Yokogawa T, Nishikawa K, Ueda T (2001). Cell-free translation reconstituted with purified components. Nat Biotechnol.

[CR36] Siltberg J, Liberles DA (2002). A simple covarion-based approach to analyse nucleotide substitution rates. J Evol Biol.

[CR37] Studer RA, Robinson-Rechavi M (2010). Large-scale analysis of orthologs and paralogs under covarion-like and constant-but-different models of amino acid evolution. Mol Biol Evol.

[CR38] Tuffley C, Steel M (1998). Modeling the covarion hypothesis of nucleotide substitution. Math Biosci.

[CR39] Wang HC, Spencer M, Susko E, Roger AJ (2007). Testing for covarion-like evolution in protein sequences. Mol Biol Evol.

[CR40] Wang HC, Susko E, Roger AJ (2011). Fast statistical tests for detecting heterotachy in Protein Evolution. Mol Biol Evol.

[CR41] Wertheim JO, Fourment M, Pond SLK (2012). Inconsistencies in estimating the age of HIV-1 subtypes due to heterotachy. Mol Biol Evol.

[CR42] Whelan S, Goldman N (2001). A general empirical model of protein evolution derived from multiple protein families using a maximum-likelihood approach. Mol Biol Evol.

[CR43] Whelan S, Blackburne BP, Spencer M (2011). Phylogenetic substitution models for detecting heterotachy during plastid evolution. Mol Biol Evol.

[CR44] Woods R, Schneider D, Winkworth CL, Riley MA, Lenski RE (2006). Tests of parallel molecular evolution in a long-term experiment with Escherichia coli. Proc Natl Acad Sci U S A.

[CR45] Wu JH, Susko E (2011). A test for heterotachy using multiple pairs of sequences. Mol Biol Evol.

[CR46] Yang Z (1994). Maximum likelihood phylogenetic estimation from DNA sequences with variable rates over sites: approximate methods. J Mol Evol.

[CR47] Yang Z, Rannala B (2012). Molecular phylogenetics: principles and practice. Nat Rev Genet.

