# Supplementary material for: Interchanging Functionality Among Homologous Elongation Factors Using Signatures of Heterotachy
Source: J Mol Evol. 2013 Jan 31;76(1):4–12. doi: 10.1007/s00239-013-9540-9 (PMC3585904; doi:10.1007/s00239-013-9540-9)
Supplement: Supplementary file 1 — Supplementary material 1 (PDF 2804 kb) [file 239_2013_9540_MOESM1_ESM.pdf]

Interchanging functionality among homologous elongation factors using signatures of heterotachy

Ercan Cacan, James T. Kratzer, Megan F. Cole and Eric A. Gaucher

Sites to disrupt eEF1B binding to eEF1A					Variant		
Yeast	Residue Number[1]	*E. coli*	Heterotachy/ Type I[2]	CBD/ Type II[3]	**KO1**	**KO2**	**KO3**
R	67	K	95.8%				
E	**68**	A	98.0%				
D	74	N	96.0%				
V	89	H	91.8%				
K	253	S	90.6%				
F	308	V	98.6%				
D	360	K	99.9%				
R	428	G	92.3%				
I	75	T	84.1%				
I	90	V	84.3%				
Y	252	F	82.9%				
N	309	L	82.7%				
K	**64**	P		59.9			
L	77	H		59.9			
T	106	A		59.9			
S	107	A		59.9			
Q	249	E		59.9			
I	257	R		59.9			
V	262	T		59.9			
H	293	F		19.1			
Q	429	R		59.9			

[1] Residue numbering is based on the sequence of yeast eEF1A (PDB: 1F60) and **bolded** sites are present in both KnockOut and KnockIn variants (See Table S2).

[2] Heterotachy/Type I sites with Posterior Probabilites (PP) listed were determined using DIVERGE's Gu99 algorithm, except those in red where DIVERGE's Gu01 algorithm was used.

[3] CBD/Type II sites (those additional sites only present in KO3) were ranked by their reported DIVERGE Type II score.

Table S1. KnockOut eEF1B binding by replacing residues in eEF1A that have a specified signature of functional divergence and are within 5 Ängstroms of eEF1B binding (PDB:1F60). In the first variant, KO1, sites having PP ≥ 90% for heterotachy/Type I were selected (8 replacements). In the second variant, KO2, sites having PP ≥ 80% for heterotachy/Type I were selected (12 replacements). In the third variant, KO3, sites having PP ≥ 80% for heterotachy/Type I plus the highest ranked CBD/Type II sites were selected (21 replacements).

Sites to introduce EF-Ts binding to eEF1A					Variant		
Yeast	Residue Number[1]	*E. coli*	Heterotachy/ Type I[2]	CBD/ Type II[3]	**KI1**	**KI2**	**KI3**
F	54	A	99.5%				
K	55	R	99.4%				
Y	56	A	99.5%				
A	57	F	96.0%				
W	58	D	95.8%				
E	**68**	A	98.0%				
I	115	V	98.6%				
V	120	D	99.9%				
D	130	M	98.3%				
S	163	E	99.9%				
Q	166	E	92.2%				
K	170	M	91.2%				
F	175	L	91.7%				
K	178	Q	99.9%				
W	194	L	90.9%				
N	346	S	95.4%				
P	348	D	91.2%				
S	394	P	96.4%				
S	18	H	84.2%				
T	23	L	84.4%				
L	60	I	82.9%				
K	129	P	82.7%				
R	164	L	83.8%				
V	179	Y	85.2%				
Q	350	C	84.8%				
K	**64**	P		59.9			
E	167	L		59.9			
G	193	A		59.9			
G	196	A		59.9			
G	349	E		59.9			
M	427	G		59.9			

[1] Residue numbering is based on the sequence of yeast eEF1A (PDB: 1F60) and **bolded** sites are present in both KnockOut and KnockIn variants (See Table S1).

[2] Heterotachy/Type I sites with Posterior Probabilites (PP) listed were determined using DIVERGE's Gu99 algorithm, except those in red where DIVERGE's Gu01 algorithm was used.

[3] CBD/Type II sites (those additional sites only present in KI3) were ranked by their reported DIVERGE Type II score.

Table S2. Knockin EF-Ts binding by replacing residues in eEF1A that have a specified signal of functional divergence and are within 5 Ängstroms of EF-Ts based on a structural alignment of PDBs 1F60 and 1EFU. In the first variant, KI1, sites having PP ≥ 90% for heterotachy/Type I were selected (18 replacements). In the second variant, KI2, sites having PP ≥ 80% for heterotachy/Type I were selected (25 replacements). In the third variant, KI3, sites having PP ≥ 80% for heterotachy/Type I plus the highest ranked CBD/Type II sites were selected (31 replacements).

Sites to disrupt eEF1B binding to eEF1A and introduce EF-Ts binding to eEF1A					Variant	
Yeast	Residue Number[1]	*E. coli*	Heterotachy/ Type I[2]	CBD/ Type II[3]	**KOKI2**	**KOKI3**
S	18	H	84.2%			
T	23	L	84.4%			
F	54	A	99.5%			
K	55	R	99.4%			
Y	56	A	99.5%			
A	57	F	96.0%			
W	58	D	95.8%			
L	60	I	82.9%			
R	67	K	95.8%			
E	**68**	A	98.0%			
D	74	N	96.0%			
I	75	T	84.1%			
V	89	H	91.8%			
I	90	V	84.3%			
I	115	V	98.6%			
V	120	D	99.9%			
K	129	P	82.7%			
D	130	M	98.3%			
S	163	E	99.9%			
R	164	L	83.8%			
Q	166	E	92.2%			
K	170	M	91.2%			
F	175	L	91.7%			
K	178	Q	99.9%			
V	179	Y	85.2%			
W	194	L	90.9%			
Y	252	F	82.9%			
K	253	S	90.6%			
F	308	V	98.6%			
N	309	L	82.7%			
N	346	S	95.4%			
P	348	D	91.2%			
Q	350	G	84.8%			
D	360	K	99.9%			
S	394	P	96.4%			
R	428	G	92.3%			
K	**64**	P		59.9		
L	77	H		59.9		
T	106	A		59.9		
S	107	A		59.9		
E	167	L		59.9		
G	193	A		59.9		
G	196	A		59.9		
Q	249	E		59.9		
I	257	R		59.9		
V	262	T		59.9		
H	293	F		19.1		
G	349	E		59.9		
M	427	G		59.9		
Q	429	R		59.9		

[1] Residue numbering is based on the sequence of yeast eEF1A (PDB: 1F60) and **bolded** sites are present in both KnockOut and KnockIn variants (See Table S1 and S2).
[2] Heterotachy/Type I sites with Posterior Probabilites (PP) listed were determined using DIVERGE's Gu99 algorithm, except those in red where DIVERGE's Gu01 algorithm was used.
[3] CBD/Type II sites (those additional sites only present in KOI3) were ranked by their reported DIVERGE Type II score.

Table S3. Combinations of eEF1B KnockOut Sites (A) and EF-Ts KnockIn Sites (B) with specified signatures of functional divergence and are within 5 Ängstroms of EF-Ts based on a structural alignment of PDBs 1F60 and 1EFU. In the first variant, KIKO2, sites having PP ≥ 80% for heterotachy/Type I were selected (36 replacements). In the second variant, KIKO3, sites having PP ≥ 80% plus the highest ranked CBD/Type II sites were selected (50 replacements).

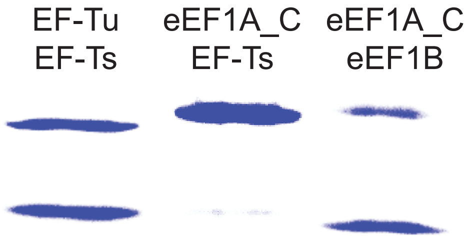

Figure S4. Control eEF1A variant binds eEF1B but not EF-Ts.
Binding assays were performed as described in Full Methods. Upper bands correspond to EF-Tu (lane 1) or eEF1A control variant (eEF1A_C, lanes 2 & 3). Lower bands correspond to bound nucleotide exchange factor (EF-Ts, lanes 1 & 2, or eEF1B, lane 3).

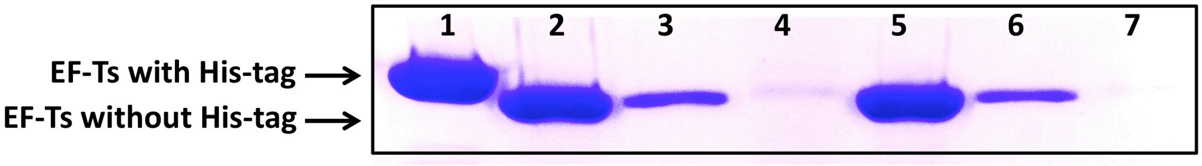

Figure S5. SDS-PAGE gel analysis of His-tag removal from EF-Ts via thrombin cleavage. EF-Ts was purified via affinity chromatography with a N-terminal 6XHis-tag on EF-Ts and passed over a Ni^{2+} column. The His-tag was removed after incubation with thrombin and the tag-less EF-Ts was passed over two additional Ni^{2+} columns to remove any non-cleaved EF-Ts still containing a His-tag. EF-Ts with His-tag (lane 1). Flow-through of thrombin-cleaved EF-Ts passed over Ni^{2+} column (lane 2), recovery of wash-step of this column (lane 3) and subsequent elution of the column (lane 4). These lanes demonstrate that thrombin cleaves the vast majority of the His-tag off EF-Ts. As further demonstration that the cleaved EF-Ts was void of His-tag, the sample from lane 2 was passed over an additional Ni^{2+} column, the flow-through was collected (lane 5), non-specific binding was removed after the wash step (lane 6), and no protein remained bound after elution (lane 7).
